# Evaluation of physicochemical and textural properties of emulsified pork model sausages treated with *Rhynchosia nulubilis* powders from different drying methods

**DOI:** 10.5713/ab.251016

**Published:** 2026-04-16

**Authors:** Min Jae Kim, Koo Bok Chin

**Affiliations:** 1 Department of Animal Science, Chonnam National University, Gwangju, Korea

**Keywords:** Emulsified Pork Model Sausage, Freeze-drying and Oven Drying, Protein Extract, *Rhynchosia nulubilis* Powder

## Abstract

**Objective:**

This study aimed to evaluate the effects of *Rhynchosia nulubilis* powder (RNP) processed by different drying methods on the physicochemical and textural properties of emulsified pork model sausages (EPMSs).

**Methods:**

RNP was prepared by freeze-drying (FP), oven-drying (OP), or obtained as a commercial powder (CP), and incorporated into EPMSs at levels of 1.0% and 3.0%. A control with no additional protein (CTL) and a reference formulation containing soy protein isolate (REF) were used for comparison. Product pH, color, water-holding capacity (cooking loss and expressible moisture), proximate composition, and texture profile parameters were evaluated.

**Results:**

RNP addition altered pH, color, water-holding capacity, and textural characteristics of EPMSs. FP- and OP-treated samples exhibited reduced cooking loss and expressible moisture compared with CTL and CP, indicating improved water-holding capacity, particularly at the 3.0% level. Redness decreased, while yellowness increased with RNP addition, depending on the processing method and concentration. Textural properties, including hardness, cohesiveness, gumminess, and chewiness, were enhanced in FP and OP treatments, whereas CP showed limited improvements.

**Conclusion:**

The results demonstrate that RNP processed by FP or OP effectively enhances the quality and functional properties of EPMSs. Properly processed RNP may serve as a promising non-meat protein ingredient for improving water retention and texture in processed meat products.

## INTRODUCTION

Emulsified meat products are widely consumed because their fat content contributes to desirable flavor and texture [1]. In particular, emulsified sausages are manufactured by forming an emulsion after finely comminuting meat and adding fat. During this process, fat acts as a key component that influences flavor, texture, and appearance, and also contributes to a feeling of satiety [2]. Moreover, fat plays an important role in emulsion stability, as well as in the emulsifying and water-holding capacities (WHC) of the product [3]. Meat proteins extracted during comminution play a vital role in stabilizing emulsified fat and retaining moisture in emulsified meat systems [4], thereby contributing to improved texture and overall product quality. However, during the heating process, protein denaturation can occur, which may increase cooking loss and consequently deteriorate the quality of the sausages [5].

To reduce cooking loss and nutrient degradation during the heating process, many studies have attempted to improve product quality by incorporating various non-meat proteins. These non-meat proteins have been reported to enhance functional properties such as WHC, emulsion stability, binding ability, and texture. Among them, soy protein isolate (SPI) is one of the most extensively studied ingredients to improve the functionality [6]; however, it has limitations due to its potential to cause soy allergies. Consequently, there is growing interest in identifying new plant-based protein sources that can replace SPI. Many other plant-derived proteins may promote the formation of protein gels during heating, thereby improving the quality and nutritional value of emulsified meat products.

*Rhynchosia nulubilis* (RN), also known as *Seomoktae* or *Yank-kong*, is characterized by its black seed coat. The seed coat of RN is rich in bioactive compounds such as the anthocyanin, which exhibit strong antioxidant activity [7]. These components have been reported to contribute to the prevention of cerebrovascular and cardiovascular diseases. In addition, RN contains high levels of amino acids and dietary fiber [8], and its protein content reaches approximately 42.5% [9], suggesting that it has potential as a protein source comparable to soybean.

Numerous studies have been conducted to utilize the high antioxidant capacity and potential functional benefits of RN [10,11], and its incorporation into food products such as cookies has been reported [12]. However, although various legume-derived proteins have been investigated in emulsified meat systems [13,14], studies on the application of RN in processed meat products remain limited. Most previous research has primarily focused on its use as a fat replacer in low-fat model sausages [15], while the functional performance of RN powder (RNP) in emulsified meat systems has not been fully evaluated. In particular, the functionality of plant protein powders can vary depending on their processing history, potentially affecting hydration behavior, solubility, and interactions with meat proteins. Previous studies have shown that drying processes can induce physicochemical modifications in plant proteins that influence their performance in food system [16]. However, the impact of such processing-dependent variations on their functionality within complex meat matrices remains poorly understood.

Drying process involves simultaneous heat and mass transfer that may induce physicochemical changes affecting the functional properties of food systems [17]. Freeze-drying is generally regarded a mild dehydration technique that preserves the porous microstructure and protein structural integrity, which can enhance the rehydration and solubility [18]. In contrast, oven-drying is a simpler and more economical process, but prolonged thermal exposure may cause structural modifications or quality deterioration by protein denaturation [19]. These processing-induced differences may influence the functional behavior of RNP in meat batter systems, although their practical effects within complex meat emulsions have not been clearly understood. Accordingly, RNPs produced under different drying conditions may exhibit variations in functional properties, potentially affecting cooking loss, WHC, and textural properties in emulsified pork model sausages (EPMSs).

Therefore, the present study aimed to investigate the applicability of RNP in EPMSs and to assess whether differences in powder processing history, including drying conditions and commercial preparation, are associated with variations in physicochemical and textural properties.

## MATERIALS AND METHODS

### Materials

Pork ham and back fat (Landrace×Yorkshire×Duroc 3-way cross-breed pig, grade 1^+^) used for preparing EPMSs were purchased from a local meat market in Korea. The whole ham was trimmed to remove connective tissues and external fat, while the back fat was cleaned to eliminate impurities. Both the trimmed ham and back fat were ground using a meat grinder (M-12S; Hankook Fuji). The trimmed meat and back fat were ground, vacuum-packed and stored at −50°C until sausage preparation.

RN used in this study was purchased from a local market (97-Farmers). The RN seeds (2 kg) were divided into two portions (1 kg each), washed, and subjected to either freeze-drying at −50°C under <8 Pa vacuum for 5 days, or oven-drying at 60°C for 7 h until constant weight was achieved. After drying, the beans were ground and sieved through a 500 μm mesh to obtain uniform powders. The freeze-dried and oven-dried powders were produced in a single batch under identical processing conditions and used throughout all experiments to minimize batch-to-batch variability. The commercial RNP was obtained from Geumwa Farming Accounting and was also passed through a 500 μm mesh to standardize particle size. The commercial powder was obtained from a single production lot; since the specific processing conditions were not disclosed, the production history of commercial RNP could not be verified. All prepared RNP samples were stored at −70°C until further use.

#### Physicochemical properties of various types of Rhynchosia nulubilis powders

The results of pH and color measurements are presented in Table 1, while the protein solubility and proximate composition results are shown in Table 2. The prepared RNPs (freeze-dried, oven-dried, and commercially obtained) were analyzed for moisture, fat, and crude protein contents (%) according to the AOAC methods [20]. The crude protein content of the powders was calculated using a nitrogen conversion factor of 6.25. Protein solubility of the RNPs was determined with slight modifications to the method described by Kim and Chin [21]. Briefly, 1 g RNP was homogenized with 14 mL of salt solution (3% salt, 17.8 mM STPP, 1 mM NaN_3_, pH 6.0) for 30 s. The homogenate was stored at 4°C for 1 h to allow protein extraction and subsequently centrifuged at 12,000×g for 30 min. The crude protein content in the supernatant was determined using the Kjeldahl method. Protein solubility was calculated as follows:


(1) Protein solubility (%)=(Protein content of supernatant)×15/Protein content of sausage batter×100

### Study I. Quality characteristics of emulsified pork model sausages containing 1.0% *Rhynchosia nulubilis* powders obtained via different drying methods and commercial powder

#### Preparation of emulsified pork model sausages added with 1.0% Rhynchosia nulubilis powders

**Figure 1:**
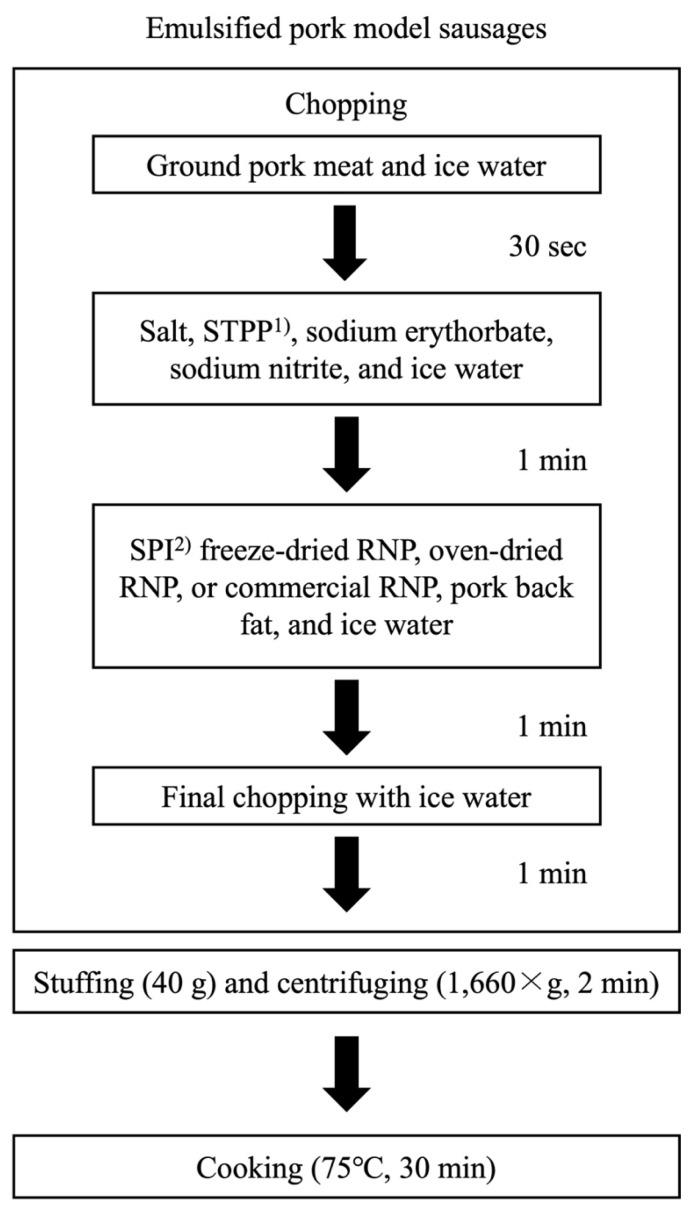
Processing of emulsified model sausages added with different types of *Rhynchosia nulubilis* powder (RNP). ^1)^ STPP: sodium tripolyphosphate. ^2)^ SPI: soybean protein isolate.

The EPMS in study 1 were prepared according to the formulation present in Table 3. To compare the quality characteristics at an equal inclusion level, SPI (SUPRO EX 33 IP) and all types of RNPs were added at 1.0%, which represents the lower range of plant protein incorporation commonly applied in commercial emulsified meat products [22]. The frozen pork meat and back fat were thawed at 4°C for 12 h prior to use, and SPI was hydrated with distilled water at a 1:4 ratio before incorporation. The detailed preparation procedure for the EPMSs is illustrated in Figure 1. The sausage batters were cooked in a water bath at 75°C for 30 min, rapidly cooled in an ice water after cooking, and then stored at 4°C until further analysis.

### Study II. Quality characteristics of emulsified pork model sausages containing 3.0% *Rhynchosia nulubilis* powders obtained via different drying methods and commercial powder

#### Preparation of emulsified pork model sausages added with 3.0% Rhynchosia nulubilis powders

The EPMSs in study 2 were prepared according to the formulation presented in Table 4. Each type of RNP was incorporated at a concentration of 3.0%. Considering that the SPI is typically used at approximately 1.5% in commercial emulsified meat formulations and contains about 90% protein, the inclusion level of RNP (with a protein content of approximately 40%, as shown in Table 2) was set at 3.0% to provide a comparable total protein contribution in the sausage formulation. As in study I, SPI was hydrated with double-distilled (dd) water at a 1:4 ratio before use, and the sausages were prepared following the formulation show in Figure 1.

#### Protein solubility

Protein solubility (%) of EPMS containing different types RNPs was determined with slight modifications to the method of Kim and Chin [21]. Briefly, 5 g of sausage batter was homogenized with 20 mL of salt solution (3% salt, 17.8 mM STPP, 1 mM NaN3, pH 6.0) for 30 s. The homogenate was stored at 4°C for 1 h to allow protein extraction and subsequently centrifuged at 12,000×g for 30 min. The crude protein content (%) of the supernatant was determined using the Kjeldahl method. Protein solubility was calculated as follows:


(2) Protein solubility (%)=(Protein content of supernatant)×5/Protein content of sausage batter×100

#### pH and color values

The pH of the cooked sausages was measured using a solid-type pH-meter (Model 340; Mettler-Toledo). Measurements were taken five times for each sample, and the average value was recorded. Color measurements were performed by cutting the cooked sausages into 1.5 cm-thick slices and evaluating the cross-sections using a Minolta Color Reader (CR-10; Minolta). The average values for lightness (L*), redness (a*), and yellowness (b*) were obtained from six measurements per sample.

#### Proximate analysis

The sausages were minced and then analyzed for proximate composition according to AOAC International [20] methods. Moisture, fat, and crude protein contents (%) were determined using the dry-oven method, Soxhlet extraction method, and Kjeldahl method, respectively. Each treatment was analyzed in duplicate, and the mean values obtained from these measurements were used for statistical analysis.

#### Cooking loss (%)

The weights of the samples before and after cooking were measured, and the difference between them was calculated and expressed as the average value using the following equation:


(3) Cooking loss (CL,%)=(Weight before cooking-Weight after cooking)/Weight before cooking×100

#### Expressible moisture (%)

Approximately 1.5 g of each sample was wrapped in three layers of filter paper and centrifuged at 1,660×g for 15 min using a centrifuge (VS-5500; Vision Science). Four samples per treatment were analyzed, and the amount of expressible moisture (EM, %) released onto the filter paper was measured. The mean value obtained from these measurements was used for analysis. The EM was calculated using the following equation:


(4) EM (%)=(Amount of moisture released onto the filter paper [g]/Sample weight [g])×100

#### Textural profile analysis

For textural profile analysis (TPA), samples were prepared into cylindrical shapes (1.25 cm in diameter and 1.3 cm in height), with ten measurements per treatment. Each sample was analyzed a Universal Testing Machine (3344; Instron) to measure hardness (gf), springiness (mm), gumminess, chewiness, and cohesiveness. The results were expressed as the mean values obtained from ten measurements.

#### Sodium dodecyl sulfate-polyacrylamide gel electrophoresis

Sodium dodecyl sulfate-polyacrylamide gel electrophoresis (SDS-PAGE) was performed using a MINI-PROTEAN 3 Cell System (Bio-Rad Laboratories). Separating and stacking gels were prepared with 10% and 4% acrylamide, respectively. Sausage batter samples collected after cooking were solubilized in 5% SDS solution. Protein concentrations of the supernatants were determined using the Pierce BCA protein assay kit (#23227; Thermo Fisher Scientific). Based on the measured protein concentrations, samples were mixed with sample buffer to obtain a final protein concentration of 1% prior to loading. Precision Plus Protein Standards (#161-0373; Bio-Rad Laboratories) were used as molecular weight markers.

#### Statistical analysis

All experiments were independently conducted three times (n = 3), and the data were analyzed using the SPSS software program (27.0; IBM). The effects of different drying methods and commercial RNPs on the physicochemical and textural properties of EPMSs were evaluated by one-way analysis of variance (ANOVA). Significant differences were determined using Duncan’s multiple range test at p<0.05.

## RESULTS AND DISCUSSION

### Study I. Quality characteristics of emulsified pork model sausages containing 1.0% *Rhynchosia nulubilis* powders obtained via different drying methods and commercial powder

#### Protein solubility

The protein solubility of EPMS batters prepared with 1.0% RNPs in EPMS is presented in Table 5. Protein solubility differed among treatments, with the freeze-dried and oven-dried RNP treatment (1.0%-FP and 1.0%-OP) showing the highest values of 48.3% and 48.7%, respectively (p<0.05). In contrast, CTL, REF, and 1.0%-CP exhibited relatively lower protein solubility values ranging from 42.8% to 44.1%, with no differences observed among these treatments (p>0.05).

Protein solubility is closely associated with functional properties such as WHC, emulsifying ability, and gel-forming ability, and salt-soluble myofibrillar proteins, including myosin and actin, play a key role in stabilizing fat droplets and forming a heat-induced protein network in meat emulsions [23,24]. Therefore, the higher protein solubility observed in 1.0%-FP and 1.0%-OP treatments may indicate improved extraction of myofibrillar proteins from the batter system, which could contribute to enhanced emulsion stability and water retention in EPMS. The higher solubility in 1.0%-FP and 1.0%-OP treatments may also be related to the physicochemical characteristics of the RNPs produced by different drying methods. Freeze-drying generally produces powders with porous structures and high surface areas [18], whereas oven drying can induce partial protein denaturation that exposes hydrophobic groups [17], potentially enhancing hydration and solubility. These characteristics may promote better dispersion of RNP proteins within the meat batter, thereby improving protein extraction efficiency. Consistent with this result, the intrinsic protein solubility of RNP was higher in FP and OP treatments (77.9% and 75.2%, respectively; Table 2) than in the CP treatment (42.0%; Table 2). In contrast, the relatively lower protein solubility observed in the CP treatment may associated with differences in the processing history of the commercial powder, which could affect protein dispersibility and extraction efficiency. Overall, increased protein solubility may facilitate the formation of a stable protein matrix during heating, contributing to improved water retention and reduced CL in EMPS.

#### pH and color values

As shown in Table 5, differences in pH and color values were observed when 1.0% of SPI and various types of RNPs were added to EPMSs. When 1.0% of RNPs were incorporated, the pH values increased compared with those of CTL and REF groups (p<0.05), whereas no differences were found among CTL, REF, and the 1.0%-CP treatment (p>0.05). However, the overall variation in pH among treatments was relatively small and therefore unlikely to cause substantial changes in product quality. The pH of meat products is influenced by both the intrinsic pH of raw meat and added ingredients, which can affect physicochemical properties such as color, texture, WHC, and gel stability. In the present study, the intrinsic pH values of the RNPs ranged from 6.91 to 7.03 and were similar among treatments (Table 1). Previous studies have reported that the addition of plant-derived proteins can increase the pH of meat products due to their relatively higher intrinsic pH compared with meat proteins [25]. However, other studies have reported negligible pH changes following the incorporation of plant proteins [1]. Consistent with these findings, a previous study on low-fat sausages also reported no differences in pH at the same RNP addition level [15]. These results suggest that the extent of pH modification in meat products may depend not only on the intrinsic pH of the added powders but also on formulation conditions, ingredient characteristics, and processing factors.

In terms of color value, the lightness (L*) values of the sausages showed no differences among the treatments (p>0.05). The CTL and REF groups exhibited the highest a* values of 9.58 and 9.16, respectively, whereas the addition of RNPs led to a decrease in a* value (p<0.05). Specifically, the a* value decreased in the order of CP>FP>OP (p<0.05), which was likely influenced by the inherent color characteristics of the RNPs. As shown in Table 1, a* values of RNPs ranged from −0.60 to −2.52, with CP exhibiting a higher a* value than the other RNPs (p<0.05). In contrast, the b* values showed an opposite trend: CTL had the lowest value, followed by REF (p<0.05). The addition of RNPs increased b* value compared to CTL and REF, with the 1.0% OP treatment showing the highest b* value among the RNP groups (p<0.05). The higher b* value of OP compared to freeze-dried RNP was likely due to browning caused by Maillard reactions under the heat conditions of oven-drying [26]. Similar changes in color parameters have been reported with the incorporation of plant-derived powders, where redness decreased and yellowness increased depending on the intrinsic pigment characteristics of the added ingredients [27].

#### Proximate analysis

The proximate composition of EPMSs containing 1.0% of different RNPs is presented in Table 5. Moisture and fat contents (%) showed no differences among treatments (p>0.05), indicating that the addition of RNPs did not affect the basic composition of the meat products. The protein content (%) of the REF group containing SPI was 14.0%, which was higher than that of CTL (p<0.05), demonstrating the role of SPI as an excellent protein source. Meanwhile, except for the 1.0% FP treatment, the sausages containing RNPs (OP and CP) showed lower protein contents than REF (p<0.05), but exhibited similar values to CTL (p>0.05). No differences in protein content (%) were observed among the RNP-added treatment (p>0.05). These results indicate that the incorporation of RNP at this level did not alter the basic composition of the sausages. The higher protein concentration of SPI compared with RNP. In contrast, no differences were observed among the RNP-added treatments, suggesting that the drying method had little influence on the proximate composition of the final products.

#### Cooking loss (%) and expressible moisture (%)

**Figure 2:**
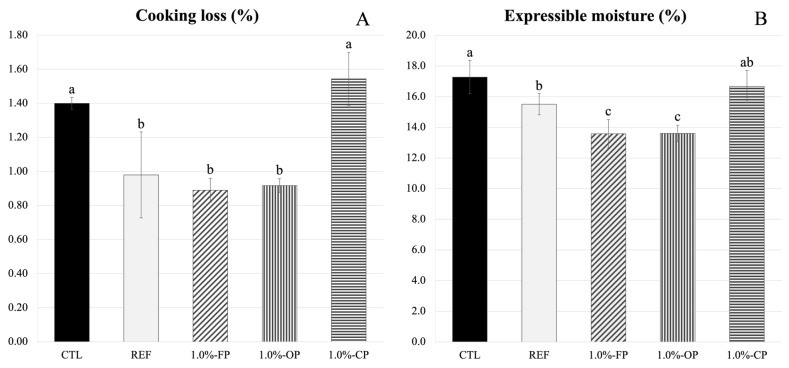
Cooking loss (CL, %) (A) and expressible moisture (EM, %) (B) of emulsified pork model sausages (EPMSs) containing 1.0% *Rhynchosia nulubilis* powder (RNP) obtained via different drying methods and commercial powder. Treatment: CTL, EPMS; REF, EPMS treated with 1.0% soy protein isolate; 1.0%-FP, EPMS treated with 1.0% freeze-dried RNP; 1.0%-OP, EPMS treated with 1.0% oven-dried RNP; 1.0%-CP, EPMS treated with 1.0% commercial RNP. ^a–c^ Means with different superscripts differ according to the various types of RNPs (p<0.05).

CL (%) is an important indicator that reflects the combined effects of protein denaturation and moisture loss during cooking and is commonly used to evaluate the WHC and overall quality of meat products. The results for CL and EM of EPMSs containing 1.0% of different RNPs are shown in Figure 2. As presented in Figure 2A, the CL values of the CTL and 1.0%-CP treatments were 1.40% and 1.54%, respectively, which were higher than those of the REF and other RNP-added treatments (1.0%-FP and 1.0%-OP) (p<0.05). The CL value of REF (0.98%) did not differ from those of the 1.0%-FP (0.89%) and 1.0%-OP (0.92%) treatments (p>0.05).

A similar trend was observed for EM (Figure 2B). The EM values of CTL (17.3%) and 1.0%-CP (16.7%) were the highest among all treatments (p<0.05), with no difference between the two groups (p>0.05). In contrast, sausages containing SPI (REF), FP, or OP exhibited lower EM values of 15.5% (REF) and 13.6% (1.0%-FP and 1.0%-OP), respectively (p<0.05). These results indicate that the incorporation of FP and OP effectively reduced moisture release, thereby improving the WHC of the meat products. The improved WHC observed in the FP and OP treatments may be associated with enhanced protein extraction and dispersion within the meat batter. As discussed in the protein solubility results, higher protein solubility was observed in 1.0%-FP and 1.0%-OP treatments, which may indicate greater extraction of salt-soluble myofibrillar proteins. These proteins play a key role in stabilizing fat droplets and forming a heat-induced gel network during cooking. Therefore, increased protein solubility may facilitate the formation of a more stable protein matrix, which helps immobilize water and fat within the system, thereby reducing CL and EM.

In contrast, the treatment containing the commercially obtained RNP (1.0%-CP) showed higher CL and EM values than the other RNP-treated groups (1.0%-FP and 1.0%-OP) (p<0.05) (Figure 2). This result may be related to differences in processing history of the commercial powder, which could influence the structural properties and dispersibility of the proteins. Reduced protein dispersibility may limit the effective interaction between plant proteins and meat proteins during batter formation, thereby weakening the protein matrix formed during heating and increasing moisture loss.

Similarly, previous studies have reported improved WHC in meat products supplemented with bean powders such as black-eyed bean, chickpea, and lentil [28]. In addition, the incorporation of common bean flour (CBF) into beef sausages was shown to enhance water retention by promoting a more stable meat protein matrix [29]. The development of such a protein network has been reported to reduce the exudation of water and fat during cooking, thereby improving the binding properties and structural stability of restructured meat products [30].

#### Textural profile analysis

The TPA results of EPMSs containing different types of RNPs are presented in Table 5. The hardness value of the 1.0%-OP treatment exhibited the highest value (3,519 gf), followed by 1.0%-FP>REF>1.0%-CP>CTL (p<0.05). For springiness, the 1.0%-FP and 1.0%-OP treatments did not differ from REF (p>0.05), but were higher than those of 1.0%-CP and CTL (p<0.05). Gumminess and chewiness were relatively higher in the 1.0%-FP and 1.0%-OP treatments compared to REF and 1.0%-CP, whereas CTL showed the lowest values (p<0.05). Cohesiveness was highest in the 1.0%-FP treatment, followed by 1.0%-OP and 1.0%-CP, while REF and CTL exhibited comparatively lower values (p<0.05).

Overall, the addition of 1.0% RNP improved most texture properties of EPMSs, particularly in the 1.0%-FP and 1.0%-OP treatments. These improvements may be associated with the formation of a more stable protein matrix during cooking. As observed in the previous results, FP- and OP-treated EPMSs exhibited higher protein solubility and improved water retention, which can promote the formation of a stronger heat-induced protein gel network and contribute to improved texture. In contrast, the treatments containing the commercial RNP (1.0%-CP) generally exhibited lower textural properties than the FP- and OP-treated EPMSs (p<0.05). This difference may be related to variations in powder characteristics that influence protein dispersibility and interactions with meat proteins during batter formation. Differences in particle size distribution or non-protein components such as dietary fiber and starch may also affect the structural development of the meat matrix. Previous studies have similarly reported that legume-derived ingredients can modify the protein matrix of meat products and influence their textural properties [29]. Instrumental hardness is generally associated with perceived with firmness during mastication; however, the relationship between instrumental texture parameters and consumer perception may vary depending on product formulation. Therefore, sensory evaluation would be required to further clarify this relationship.

#### Sodium dodecyl sulfate-polyacrylamide gel electrophoresis

The SDS-PAGE patterns of EPMS containing 1.0% RNPs are shown in Figure 4. In all treatments, bands corresponding to myosin heavy chain (MHC) were observed around 250 kDa, actin bands were detected in the range of 37–50 kDa, and myosin light chain (MLC) bands appeared around 20 kDa. In addition, additional bands were observed around 75 kDa and 50 kDa in the RNP-containing treatments. According to Keum et al [31], these bands correspond to the α′, α, and β subunits of 7S globulin, a major storage protein in legume. Similar bands were also reported in low-fat model sausages containing RNP [15]. These results indicate that RNP-derived proteins were incorporated into the protein matrix of the meat product. The bands were more distinct in 1.0%-FP and 1.0%-OP treatments, suggesting that RNP proteins were dispersed within meat protein system. Jiang and Xiong [32] reported that β-conglycinin and glycinin, the major components of SPI, interact with myosin to promote gel formation and improve gel strength. In addition, a high-molecular-weight band observed at the top of the gel likely represents protein aggregates formed through protein-protein interactions during heating. These results suggest that RNP proteins may participate in the formation of the protein network together with meat proteins during thermal processing.

### Study II. Quality characteristics of emulsified pork model sausages containing 3.0% *Rhynchosia nulubilis* powders obtained via different drying methods and commercial powder

#### Protein solubility

The protein solubility of EPMS batters containing 3.0% RNPs is presented in Table 6. The CTL showed the lowest protein solubility (40.7%) (p<0.05), whereas the REF, 3.0%-FP, 3.0%-OP, and 3.0%-CP treatments exhibited higher values ranging from 46.2% to 49.0% compared with the CTL (p<0.05). However, no differences were observed among the SPI and RNP-added treatments (p>0.05). The relatively higher protein solubility observed in 3.0%-FP and 3.0%-OP treatments showed a similar tendency to that observed at the 1.0% addition level (Table 5). However, at the 3.0% addition level, no differences were observed among the FP, OP, and CP treatments, suggesting that the influence of protein supplementation itself became more dominant than differences in powder characteristics. The increase in protein solubility observed in the RNP-added treatments may be associated with enhanced extraction of salt-soluble proteins in the batter system. Salt-soluble myofibrillar proteins, such as myosin and actin, play a key role in stabilizing emulsified meat systems by forming a protein matrix during cooking. The addition of plant-derived proteins may facilitate the dispersion and interactions with meat proteins, thereby promoting protein extraction. Similar results have been reported for SPI-added meat batters, where improved solubility of myofibrillar proteins was attributed to interactions between soy proteins and actomyosin [25,33]. These results suggest that differences in powder characteristics may be more evident at lower addition levels, whereas at higher incorporation levels the overall protein contribution on the added ingredient becomes the dominant factor affecting protein solubility.

#### pH and color values

The pH values of EPMSs containing 3.0% of different RNPs are presented in Table 6. The pH ranged from 6.28 to 6.37 and differed depending on the type of RNP added (p<0.05). The CTL and 3.0%-CP treatments exhibited the lowest pH values (6.28 and 6.29, respectively), whereas REF and 3.0%-FP showed the highest values (6.37 and 6.36, respectively) (p<0.05). When RNPs excluding CP were incorporated, the pH values were higher than that of CTL, but lower than that of REF (p<0.05). It has been reported that the addition of SPI to processed meat products can increase the final product’s pH due to the inherently high pH of SPI as compared to meat [25]. Similarly, the elevated pH observed in 3.0%-FP and 3.0%-OP treatments compared with CTL likely reflects the higher pH values of FP and OP powders themselves, which were 7.00 and 7.01, respectively (Table 1). Kandil et al [34] also reported that partial replacement of fat with chickpea protein isolate (CPI) in beef sausages increased pH compared with the control. In contrast, Pietrasik and Janz [35] found no pH change were observed when pea flour was added to bologna sausages. de Souza Paglarini et al [1] reported no pH change were observed when emulsion gels prepared with SPI were used as partial fat replacers. In contrast, the CP treatment showed a pH value similar to that of CTL, suggesting that differences in powder characteristics may influence the pH response in the sausage system.

The color values (L*, a*, and b*) of EPMSs containing 3.0% of different RNPs are presented in Table 6. The CTL and REF groups showed similar L* and a* values (p>0.05), whereas the addition of RNPs resulted in decreased L* and a* across treatments (p<0.05). In particular, the 3.0%-FP treatment exhibited the lowest a* value (3.93) among all groups (p<0.05). Compared to a* value, an opposite trend was observed for b* values. The CTL had the lowest b* value (6.97), while the 3.0%-CP treatment exhibited the highest value (10.1) (p<0.05). The b* values of 3.0%-FP and 3.0%-OP were higher than those of CTL (p<0.05), but did not differ from REF (p>0.05). These results are likely attributable to the inherent color characteristics of the legume powders added to the EPMSs. As shown in Table 1, SPI had a higher a* value (1.12) than all RNPs (FP: −2.45; OP: −2.52; CP: −0.60), whereas the RNPs exhibited higher b* values than SPI. Chin et al [36] reported that the addition of SPI to bologna sausages decreased redness, which was attributed to a relative reduction in myoglobin content due to the inclusion of non-meat proteins [37]. Similarly, in this study, the addition of RNPs resulted in decreased redness, consistent with previous findings that non-meat proteins can influence meat color by diluting myoglobin concentration.

#### Proximate analysis

The proximate composition of EPMSs containing 3.0% of different RNPs is presented in Table 6. According to the results, no differences were observed among treatments in moisture, fat, and protein contents (p>0.05), indicating that the addition of RNPs did not affect the proximate composition of the EPMSs. The moisture contents in all treatments ranged from 61.6% to 62.7%, with no statistical differences between treatments (p>0.05). Fat contents from 20.6% to 22.0%, showing similar values across all groups (p>0.05), suggesting that the RNPs used in this study did not influence fat retention in the product. The protein content in all treatments ranged from 13.4% to 14.2%, which was not statistically significant (p>0.05). These results suggest that the incorporation of RNP at this level did not alter the proximate composition of the sausages.

#### Cooking loss (%) and expressible moisture (%)

**Figure 3:**
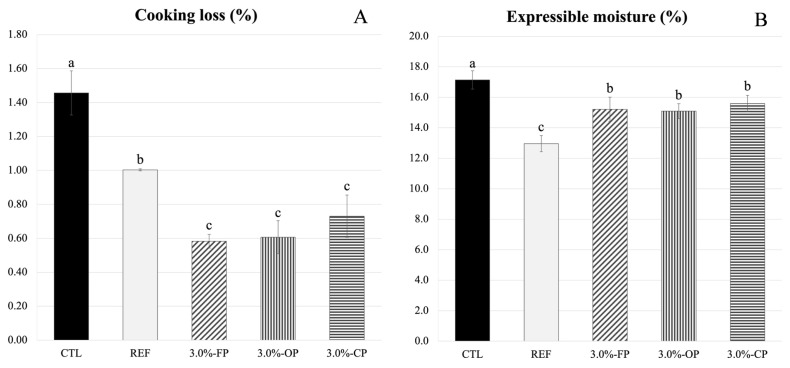
Cooking loss (CL, %) (A) and expressible moisture (EM, %) (B) of emulsified pork model sausages (EPMSs) containing 3.0% *Rhynchosia nulubilis* powder (RNP) obtained via different drying methods and commercial powder. Treatment: CTL, EPMS; REF, EPMS treated with 1.5% soy protein isolate; 3.0%-FP, EPMS treated with 3.0% freeze-dried RNP; 3.0%-OP, EPMS treated with 3.0% oven-dried RNP; 3.0%-CP, EPMS treated with 3.0% commercial RNP. ^a–c^ Means with different superscripts differ according to the various types of RNPs (p<0.05).

The results of CL (%) and EM (%) for EPMSs containing 3.0% of different RNPs are presented in Figure 3. CL represents the proportion of moisture and fat lost during cooking and is closely related to the WHC of the product. In this study, CTL exhibited the highest CL value (1.46%) among all treatments (p<0.05), while REF showed a lower value of 1%. When various RNPs were added, CL values ranged from 0.58% to 0.73%, which were lower than those of CTL and REF (p<0.05). The reduction of CL observed in the RNP-added EPMSs is likely related to the water absorption and gel-forming abilities of non-meat proteins and dietary fiber. These components can absorb water within the gel matrix during cooking and interact with meat proteins to form a stable 3D network, thereby enhancing water retention in the product [37]. Similarly, Argel et al [38] reported that the addition of white bean powder (8–15 g/kg) to pork burger patties decreased CL, and Kim and Chin [13] demonstrated that the incorporation of faba bean protein isolate (FBPI) into pork sausages reduced CL regardless of inclusion level. These findings support that the addition of legume-derived proteins, such as RNP, can enhance water retention and minimize cooking-induced losses in emulsified meat systems.

WHC is an important indicator of both the economic and sensory quality of meat products, as higher WHC is associated with reduced CL and improved juiciness. As shown in Figure 3, the EM value of CTL was the highest (17.1%), whereas REF showed the lowest EM value (13.0%) (p<0.05). The EM values of EPMSs containing 3.0% of different RNPs were lower than that of CTL (p<0.05), indicating that RNP addition contributed to the improvement of WHC. According to Shen et al [39], the incorporation of pea protein isolate (PPI) into beef patties enhanced WHC by forming a gel matrix during cooking that improved water absorption and retention. In the present study, the reduction in EM following RNP addition supports the potential of RNP as a functional ingredient for improving WHC in meat products. In contrast, in Study I (1.0% addition), the CP treatment showed CL and EM values comparable to CTL, indicating minimal improvement in WHC. However, in study II (3.0% addition), the CP treatment showed no differences from FP and OP. This suggests that increasing the inclusion level may partially enhance WHC, although the functionality of the commercial RNP may be limited at low concentrations.

#### Textural profile analysis

The TPA of EPMSs containing 3.0% of different RNPs is presented in Table 6. The hardness values of the FP and OP treatments were comparable to those of REF (p>0.05), suggesting that freeze-dried and oven-dried RNPs positively contributed to heat-induced gel formation between meat proteins. In contrast, the 3.0%-CP treatment showed a lower hardness value than REF (p<0.05) and was similar to CTL (p>0.05), indicating no improvement in texture. This could be attributed to factors such as protein denaturation or oxidation, particle size irregularity, and heat-stabilized protein structure possibly occurring during the commercial processing and distribution of CP. These factors may have interfered with protein-protein interactions and gel network formation, resulting in limited enhancement of textural properties. Similar trends were observed in other textural characteristics including springiness, gumminess, and chewiness. The FP and OP treatments showed higher values than CTL, indicating improved textural characteristics, while springiness was highest in REF (p<0.05). Among the RNP treatments, springiness ranged from 4.48 to 4.69 mm, with no differences among them (p>0.05). REF exhibited the highest gumminess and chewiness values (p<0.05), which were similar to those of the 3.0%-OP and 3.0%-CP treatments but were higher than those of CTL and 3.0%-FP (p<0.05). Overall, RNP-added samples showed texture characteristics comparable to those of REF (p<0.05), except for the freeze-dried treatment. Therefore, the incorporation of 3.0% RNP effectively improved the textural properties of EPMSs compared with CTL; however, the enhancement observed in the 3.0%-CP treatment was relatively limited. Notably, the textural properties of FP- and OP-treated samples were comparable to those of REF formulated with SPI, suggesting that RNP produced through controlled drying processes could potentially serve as an alternative plant-derived functional ingredient in emulsified meat products.

#### Sodium dodecyl sulfate-polyacrylamide gel electrophoresis

**Figure 4:**
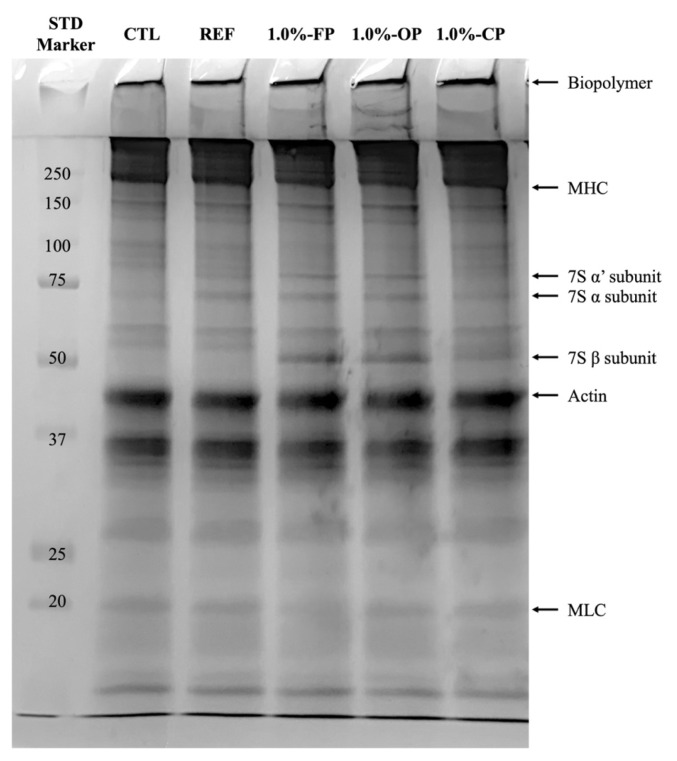
SDS-PAGE patterns of emulsified pork model sausages (EPMSs) containing 1.0% *Rhynchosia nulubilis* powder (RNP) obtained via different drying methods and commercial powder. STD Marker: standard marker; Treatment: CTL, EPMS; REF, EPMS treated with 1.0% soy protein isolate; 1.0%-FP, EPMS treated with 1.0% freeze-dried RNP; 1.0%-OP, EPMS treated with 1.0% oven-dried RNP; 1.0%-CP, EPMS treated with 1.0% commercial RNP. MHC, myosin heavy chain; MLC, myosin light chain; SDS-PAGE, sodium dodecyl sulfate-polyacrylamide gel electrophoresis.

**Figure 5:**
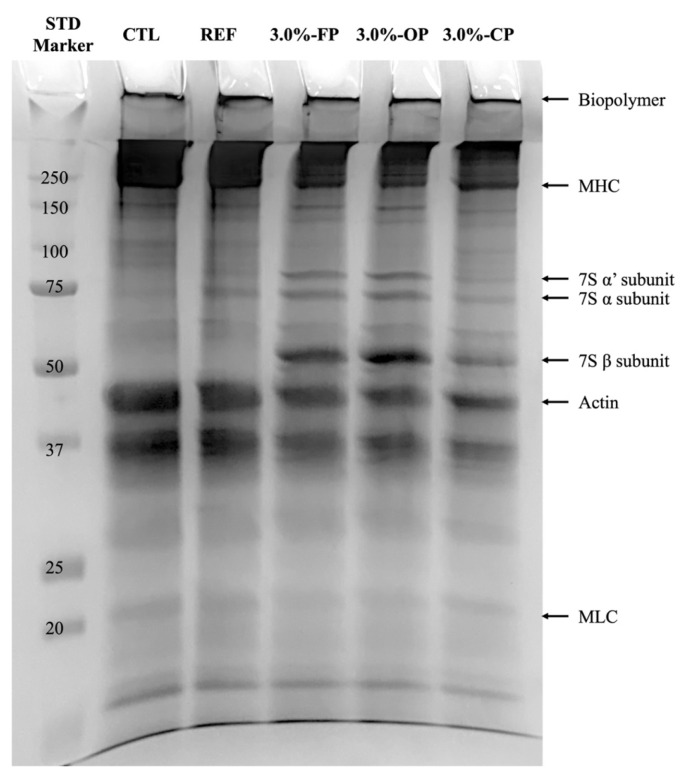
SDS-PAGE patterns of emulsified pork model sausages (EPMSs) containing 3.0% *Rhynchosia nulubilis* powder (RNP) obtained via different drying methods and commercial powder. STD Marker: standard marker; Treatment: CTL, EPMS; REF, EPMS treated with 1.5% soy protein isolate; 3.0%-FP, EPMS treated with 3.0% freeze-dried RNP; 3.0%-OP, EPMS treated with 3.0% oven-dried RNP; 3.0%-CP, EPMS treated with 3.0% commercial RNP. MHC, myosin heavy chain; MLC, myosin light chain; SDS-PAGE, sodium dodecyl sulfate-polyacrylamide gel electrophoresis.

The SDS-PAGE patterns of EPMS containing 3.0% RNPs are shown in Figure 5. In all treatments, the major myofibrillar protein bands corresponding to MHC (approximately 250 kDa), actin (37–50 kDa), and MLC (approximately 20 kDa) were observed, and the overall protein patterns were similar to those observed at the 1.0% addition level (Figure 4). As in Figure 4, bands corresponding to RNP-derived proteins, identified as 7S globulin subunits (approximately 75 kDa: α′ subunit and α subunit; approximately 50 kDa: β subunit), were detected in the RNP-added treatments. However, the intensity of the 7S globulin bands in the CP treatment was relatively lower than that observed in the other RNP-added treatments, suggesting that differences in processing history or protein characteristics of the powders may have influenced the distribution of proteins within the meat product system. Meanwhile, a biopolymer band, presumed to be a high-molecular weight protein aggregate, was observed at the top of the stacking gel. This band is considered to represent a protein complex formed through protein-protein interactions during the heating process. However, no noticeable differences in these high-molecular weight protein bands were observed among treatments. These results suggest that RNP proteins may coexist with meat protein matrix and partially contribute to the formation of the protein network during heating.

## CONCLUSION

This study evaluated the effects of RNP produced by different drying methods (freeze-drying and oven-drying), as well as a commercially available powder, on the physicochemical and textural properties of EPMSs. The incorporation of RNP, except for the commercial powder, improved protein solubility, WHC (CL and EM), and textural properties of the sausages. At the 1.0% addition level, freeze-dried and oven-dried RNP exhibited superior functional performance compared with the commercial powder, showing lower CL and improved texture. At the 3.0% addition level, the addition of RNP enhanced protein solubility and WHC regardless of powder type, indicating that the effect of protein incorporation was more pronounced than the differences among powder types. SDS-PAGE analysis suggested that RNP-derived proteins participated in the meat protein matrix. Based on these results, an additional level of approximately 1.0% RNP may be considered optimal for improving the physicochemical and textural properties of EMPSs while maintaining formulation stability. Overall, RNP may serve as a potential plant-derived functional ingredient for improving the quality of emulsified meat products and may provide a feasible alternative to conventional plant protein ingredients such as SPI in industrial meat formulations. Further studies are needed to evaluate the applicability of RNP under practical meat processing conditions and to investigate its sensory characteristics.

## Figures and Tables

**Table 1:** pH and color values of SPI and *Rhynchosia nulubilis* powder (RNP) obtained via different drying methods and commercial powder

	Treatment^1)^			

	SPI	FP	OP	CP
pH	7.03±0.01^a^	7.00±0.00^c^	7.01±0.00^b^	6.91±0.01^d^
CIE L* (lightness)	87.4±0.35^a^	80.6±0.20^c^	81.9±0.84^b^	78.9±0.71^d^
CIE a* (redness)	1.12±0.19^a^	−2.45±0.00^c^	−2.52±0.38^c^	−0.60±0.05^b^
CIE b* (yellowness)	13.6±0.08^d^	15.1±0.40^c^	15.7±0.31^b^	18.4±0.03^a^

1) Treatment: SPI, soy protein isolate; FP, freeze-dried RNP; OP, oven-dried RNP; CP, commercial RNP.

a–d Means with different superscripts differ according to the various types of RNPs (p<0.05).

**Table 2:** Protein solubility, proximate analysis of *Rhynchosia nulubilis* powder (RNP) obtained via different drying methods and commercial powder

	Treatment^1)^		

	FP	OP	CP
Protein solubility (%)	77.9±1.68^a^	75.2±2.26^a^	42.0±0.93^b^
Moisture (%)	6.54±0.15^a^	6.58±0.13^a^	4.84±0.16^b^
Fat (%)	15.7±1.34^a^	16.0±1.90^a^	13.9±0.35^b^
Protein (%)	40.2±1.50^a^	41.1±0.57^a^	40.8±1.13^a^

1) Treatment: FP, freeze-dried RNP; OP, oven-dried RNP; CP, commercial RNP.

a,b Means with different superscripts differ according to the various types of RNPs (p<0.05).

**Table 3:** The formulation of emulsified pork model sausages (EPMSs) containing 1.0% *Rhynchosia nulubilis* powder (RNP) obtained via different drying methods and commercial powder

Ingredient (%)	Treatment^1)^				

	CTL	REF	1.0%-FP	1.0%-OP	1.0%-CP
1. Meat	60.00	60.00	60.00	60.00	60.00
2. Fat	20.00	20.00	20.00	20.00	20.00
3. Water	18.13	18.13	18.13	18.13	18.13
^1)^ Ice water	18.13	14.13	18.13	18.13	18.13
2) Hydrated water	0.00	4.00	0.00	0.00	0.00
4. Non-meat ingredients	1.87	2.87	2.87	2.87	2.87
^1)^ Salt	1.50	1.50	1.50	1.50	1.50
2) STPP	0.30	0.30	0.30	0.30	0.30
3) Sodium erythorbate	0.05	0.05	0.05	0.05	0.05
4) Sodium nitrite	0.015	0.015	0.015	0.015	0.015
5) Soy protein isolate	0.00	1.00	0.00	0.00	0.00
6) RNP	0.00	0.00	1.00	1.00	1.00
(^1)^ Freeze-drying	0.00	0.00	1.00	0.00	0.00
(2) Oven-drying	0.00	0.00	0.00	1.00	0.00
(3) Commercial	0.00	0.00	0.00	0.00	1.00
Total	100.00	101.00	101.00	101.00	101.00

1) Treatment: CTL, EPMS; REF, EPMS treated with 1.0% soy protein isolate; 1.0%-FP, EPMS treated with 1.0% freeze-dried RNP; 1.0%-OP, EPMS treated with 1.0% oven-dried RNP; 1.0%-CP, EPMS treated with 1.0% commercial RNP.

STPP, sodium tripolyphosphate.

**Table 4:** The formulation of emulsified pork model sausages (EPMSs) containing 3.0% *Rhynchosia nulubilis* powder (RNP) obtained via different drying methods and commercial powder

Ingredient (%)	Treatment^1)^				

	CTL	REF	3.0%-FP	3.0%-OP	3.0%-CP
1. Meat	60.00	60.00	60.00	60.00	60.00
2. Fat	20.00	20.00	20.00	20.00	20.00
3. Water	18.13	18.13	18.13	18.13	18.13
^1)^ Ice water	18.13	12.13	18.13	18.13	18.13
2) Hydrated water	0.00	6.00	0.00	0.00	0.00
4. Non-meat ingredients	1.87	3.37	4.87	4.87	4.87
^1)^ Salt	1.50	1.50	1.50	1.50	1.50
2) STPP	0.30	0.30	0.30	0.30	0.30
3) Sodium erythorbate	0.05	0.05	0.05	0.05	0.05
4) Sodium nitrite	0.015	0.015	0.015	0.015	0.015
5) Soy protein isolate	0.00	1.50	0.00	0.00	0.00
6) RNP	0.00	0.00	3.00	3.00	3.00
(^1)^ Freeze-drying	0.00	0.00	3.00	0.00	0.00
(2) Oven-drying	0.00	0.00	0.00	3.00	0.00
(3) Commercial	0.00	0.00	0.00	0.00	3.00
Total	100.00	101.50	103.00	103.00	103.00

1) Treatment: CTL, EPMS; REF, EPMS treated with 1.5% soy protein isolate; 3.0%-FP, EPMS treated with 3.0% freeze-dried RNP; 3.0%-OP, EPMS treated with 3.0% oven-dried RNP; 3.0%-CP, EPMS treated with 3.0% commercial RNP.

STPP, sodium tripolyphosphate.

**Table 5:** Physicochemical and textural properties of emulsified pork model sausages (EPMSs) containing 1.0% *Rhynchosia nulubilis* powder (RNP) obtained via different drying methods and commercial powder

	Treatment^1)^				

	CTL	REF	1.0%-FP	1.0%-OP	1.0%-CP
Protein solubility (%)	43.3±1.11^b^	44.1±0.76^b^	48.3±0.43^a^	48.7±0.69^a^	42.8±1.71^b^
pH	6.00±0.06^b^	6.00±0.07^b^	6.15±0.02^a^	6.16±0.01^a^	6.00±0.06^b^
CIE L* (lightness)	71.1±1.34^a^	71.9±0.56^a^	70.6±0.83^a^	71.7±0.82^a^	70.1±0.99^a^
CIE a* (redness)	9.58±0.53^a^	9.16±0.12^a^	5.84±0.08^c^	4.97±0.20^d^	8.09±0.08^b^
CIE b* (yellowness)	7.51±0.22^d^	7.87±0.11^c^	8.51±0.04^b^	9.06±0.10^a^	8.74±0.17^b^
Moisture (%)	64.5±0.74^a^	63.6±0.26^a^	63.2±0.95^a^	63.3±0.69^a^	64.4±0.75^a^
Fat (%)	20.5±0.86^a^	19.5±1.12^a^	20.4±1.95^a^	21.0±1.78^a^	20.7±0.80^a^
Protein (%)	12.1±0.94^c^	14.0±0.38^a^	13.2±0.11^ab^	13.0±0.27^bc^	12.9±0.45^bc^
Hardness (gf)	2,471±54.3^e^	3,167±54.6^c^	3,322±59.8^b^	3,519±23.3^a^	2,960±130^d^
Springiness (mm)	4.00±0.04^bc^	4.17±0.06^a^	4.10±0.01^ab^	4.13±0.01^ab^	3.93±0.14^c^
Gumminess	20.6±0.47^c^	28.3±0.68^b^	38.4±1.63^a^	36.6±1.13^a^	29.6±2.71^b^
Chewiness	82.4±1.11^c^	118±3.09^b^	157±6.19^a^	151±4.21^a^	116±6.72^b^
Cohesiveness	0.84±0.01^d^	0.89±0.01^c^	1.15±0.03^a^	1.04±0.03^b^	1.00±0.05^b^

1) Treatment: CTL, EPMS; REF, EPMS treated with 1.0% soy protein isolate; 1.0%-FP, EPMS treated with 1.0% freeze-dried RNP; 1.0%-OP, EPMS treated with 1.0% oven-dried RNP; 1.0%-CP, EPMS treated with 1.0% commercial RNP.

a–e Means with different superscripts differ according to the various types of RNPs (p<0.05).

**Table 6:** Physicochemical and textural properties of emulsified pork model sausages (EPMSs) containing 3.0% *Rhynchosia nulubilis* powder (RNP) obtained via different drying methods and commercial powder

	Treatment^1)^				

	CTL	REF	3.0%-FP	3.0%-OP	3.0%-CP
Protein solubility (%)	40.7±1.75^b^	46.2±1.26^a^	48.8±2.13^a^	49.0±1.23^a^	46.4±0.41^a^
pH	6.28±0.02^c^	6.37±0.01^a^	6.36±0.01^a^	6.33±0.01^b^	6.29±0.02^c^
CIE L* (lightness)	70.1±0.95^a^	69.8±1.15^a^	67.4±0.67^b^	68.3±1.46^ab^	66.6±0.73^b^
CIE a* (redness)	9.34±0.32^a^	9.18±0.48^a^	3.93±0.15^d^	4.70±0.30^c^	5.82±0.33^b^
CIE b* (yellowness)	6.97±0.33^c^	7.95±0.33^b^	7.90±0.40^b^	8.22±0.40^b^	10.1±0.34^a^
Moisture (%)	62.6±0.95^a^	62.7±0.64^a^	62.0±0.92^a^	62.0±0.75^a^	61.6±1.46^a^
Fat (%)	22.0±0.88^a^	21.3±0.73^a^	20.6±1.52^a^	20.7±1.34^a^	21.4±1.88^a^
Protein (%)	13.4±0.54^a^	13.4±0.35^a^	13.9±0.60^a^	14.2±0.22^a^	13.7±0.16^a^
Hardness (gf)	3,090±202^c^	3,614±227^a^	3,408±144^ab^	3,442±106^ab^	3,228±62.9^bc^
Springiness (mm)	4.37±0.20^b^	4.99±0.33^a^	4.48±0.17^b^	4.69±0.12^ab^	4.60±0.13^ab^
Gumminess	26.9±2.46^c^	35.9±3.20^a^	31.2±1.02^b^	31.5±0.94^b^	30.2±0.39^bc^
Chewiness	117±11.4^c^	179±18.8^a^	140±7.14^b^	148±8.06^b^	139±4.50^b^
Cohesiveness	0.87±0.03^c^	0.99±0.04^a^	0.92±0.02^bc^	0.91±0.01^bc^	0.94±0.02^b^

1) Treatment: CTL, EPMS; REF, EPMS treated with 1.5% soy protein isolate; 3.0%-FP, EPMS treated with 3.0% freeze-dried RNP; 3.0%-OP, EPMS treated with 3.0% oven-dried RNP; 3.0%-CP, EPMS treated with 3.0% commercial RNP.

a–d Means with different superscripts differ according to the various types of RNPs (p<0.05).

## Data Availability

Upon reasonable request, the datasets of this study can be available from the corresponding author.
